# Mitochondrial Bioenergetics in the Metabolic Myopathy Accompanying Peripheral Artery Disease

**DOI:** 10.3389/fphys.2017.00141

**Published:** 2017-03-13

**Authors:** Victoria G. Rontoyanni, Omar Nunez Lopez, Grant T. Fankhauser, Zulfiqar F. Cheema, Blake B. Rasmussen, Craig Porter

**Affiliations:** ^1^Metabolism Unit, Shriners Hospitals for ChildrenGalveston, TX, USA; ^2^Department of Surgery, University of Texas Medical BranchGalveston, TX, USA; ^3^Department of Nutrition and Metabolism, University of Texas Medical BranchGalveston, TX, USA

**Keywords:** mitochondria, mitochondrial function, bioenergetics, peripheral artery disease, peripheral vascular disease, skeletal muscle

## Abstract

Peripheral artery disease (PAD) is a serious but relatively underdiagnosed and undertreated clinical condition associated with a marked reduction in functional capacity and a heightened risk of morbidity and mortality. The pathophysiology of lower extremity PAD is complex, and extends beyond the atherosclerotic arterial occlusion and subsequent mismatch between oxygen demand and delivery to skeletal muscle mitochondria. In this review, we evaluate and summarize the available evidence implicating mitochondria in the metabolic myopathy that accompanies PAD. Following a short discussion of the available *in vivo* and *in vitro* methodologies to quantitate indices of muscle mitochondrial function, we review the current evidence implicating skeletal muscle mitochondrial dysfunction in the pathophysiology of PAD myopathy, while attempting to highlight questions that remain unanswered. Given the rising prevalence of PAD, the detriment in quality of life for patients, and the associated significant healthcare resource utilization, new alternate therapies that ameliorate lower limb symptoms and the functional impairment associated with PAD are needed. A clear understanding of the role of mitochondria in the pathophysiology of PAD may contribute to the development of novel therapeutic interventions.

## Introduction

Peripheral artery disease (PAD) is the third leading cause of cardiovascular morbidity after coronary heart disease and stroke (Fowkes et al., 2013). In the preceding decade, the global prevalence of PAD increased by ~24%, corresponding to an estimated 202 million people living with PAD worldwide (Fowkes et al., 2013). In the US alone, ~8.5 million adults aged ≥40 years are affected by PAD (Allison et al., 2007; Mozaffarian et al., 2015), with annual hospitalization costs estimated at more than $21 billion (Mahoney et al., 2008, 2010). Yet, diagnosis and treatment of PAD is often overlooked, and clinical trials focusing on PAD are relatively sparse considering the prevalence and impact of the disease (Jaff, 2014; Subherwal et al., 2014).

As an atherosclerotic occlusive disease, PAD results in obstruction of the conduit arteries serving the lower extremities. Reduced blood flow, and skeletal muscle and nerve abnormalities contribute to the functional impairment and limb manifestations associated with PAD. In primary care facilities, 30–60% of all PAD patients are diagnosed as asymptomatic, (i.e., no exertional leg symptoms), ~10% exhibit classic symptoms of intermittent claudication (i.e., exertional calf pain that is induced by exercise/walking and subsides with rest), and the remainder present with atypical leg symptoms, i.e., exertional leg symptoms that are not consistent with classic intermittent claudication. Atypical leg symptoms commonly include leg pain on exertion and rest (i.e., exertional leg pain that initiates at rest but is not associated with critical limb ischemia), and leg pain/carry on (i.e., exertional leg pain that does not prompt the patient to stop exercising/walking; McDermott, 2015; Hiatt et al., 2015). Over a 5-year follow-up, 1–2% of PAD cases will progress to critical limb ischemia (i.e., ischemic rest pain and tissue loss, such as skin ulceration and gangrene; Hirsch et al., 2006; McDermott, 2015). Irrespective of this classification, all patients with PAD, even asymptomatic individuals, have reduced functional capacity (McDermott et al., 2000, 2008) and a heightened risk of morbidity and mortality (Diehm et al., 2009). Over time, functional capacity in PAD declines further, the degree of the change being associated with the severity of the disease (McDermott et al., 2004).

Individuals with PAD are less physically active (McDermott et al., 2002) and tend to have a maximal exercise capacity that is approximately half of that of age-matched healthy individuals (Hiatt et al., 1987). Further, patients with PAD who develop critical limb ischemia are at increased risk for limb loss. Strikingly, PAD accounts for ~70,000 major amputations performed annually within the U.S., with an estimated cost of $10.6 billion (Yost, 2014). It is predicted that the number of all-cause amputees living in the US will rise from 1.6 million to 3.6 million by 2,050, with PAD accounting for the majority of this projected increase (Ziegler-Graham et al., 2008). Moreover, PAD patients who require lower limb amputation have a 5-year mortality of over 50%, a poorer prognosis to that of breast, prostate, and colon cancer patients (Armstrong et al., 2007; Robbins et al., 2008). Thus, PAD represents a significant problem in modern health care, in terms of its increasing prevalence, fiscal impact on health care systems, and detriment in quality of life for patients.

Considering the largely asymptomatic nature of PAD in its early stages (McDermott et al., 2000; Hirsch et al., 2006; McDermott, 2015), and the detrimental consequences of its progression, early clinical diagnosis, and effective management of PAD is imperative. The most cost-effective tool for early clinical diagnosis of lower extremity PAD is the ankle-brachial index (ABI), which is the ratio of resting systolic blood pressure measured at the ankle (dorsalis pedis and posterior tibial arteries) to that at the brachial artery; an ABI value of <0.90 is 68–84% sensitive and 84–99% specific for PAD (Aboyans et al., 2012; Gerhard-Herman et al., 2016). Major risk factors for PAD include advancing age, hypertension, smoking, diabetes mellitus, and hyperlipidemia (Fowkes et al., 2013). As with other forms of atherosclerotic vascular disease, the 2016 AHA/ACC guidelines for treatment of PAD recommend cardiovascular risk factor modification, in addition to PAD specific treatment for claudication and critical limb ischemia. Claudication is primarily addressed via exercise training, and administration of phosphodiesterase inhibitors for their antiplatelet and vasodilatory properties (Hirsch et al., 2006; Gerhard-Herman et al., 2016).

Effective long-term management of limb symptomatology and functional impairment in PAD depends on targeted therapies addressing the pathophysiology of the disease. While atherosclerotic occlusive disease, decreased blood perfusion, and restricted O_2_ delivery to skeletal muscle is a major contributor to the limb manifestations in PAD, reduced blood flow only partially accounts for the functional deficit associated with PAD. Additional factors contribute to the pathophysiology of the functional impairment in PAD, with evidence suggesting abnormalities in skeletal muscle metabolism, where mitochondria may play a key role (Brass and Hiatt, 2000; Kemp, 2004; Pipinos et al., 2007). Accordingly, the aim of this review article is to explore and discuss the current evidence for a role of mitochondrial dysfunction in the metabolic myopathy observed in PAD patients. Given the rising prevalence of PAD and the societal impact that it incurs, a better understanding of the underlying determinants of reduced muscle function seen in PAD patients represents a critical step in the development of novel therapeutic interventions.

## Pathophysiology of limb manifestations and functional impairment in PAD

### Hemodynamic abnormalities

The sequelae of events leading to limited functional capacity in PAD patients originate from a hemodynamic deficit resulting from one or more arterial stenoses and/or occlusions in the iliac, femoral, popliteal, or tibial arteries (Hiatt et al., 2015). Blood flow distribution to tissues is relative to their metabolic activity. At rest, skeletal muscle perfusion represents ~20% of cardiac output (McArdle et al., 2007). During exercise, blood flow is redistributed to the active skeletal muscles, with muscle blood flow accounting for up to 80–85% of cardiac output at high exercise intensities (McArdle et al., 2007). Although the presence of arterial stenosis or occlusion can adversely affect blood flow dynamics, in the absence of critical limb ischemia resting blood flow to lower extremities is preserved via compensatory mechanisms distal to the site of the occlusion, such as collateral blood supply (Bragadeesh et al., 2005; Traupe et al., 2013). However, in states of high metabolic demand, such as exercise, the arterial stenosis, and occlusions in PAD become flow limiting. Blood flow is further compromised by impaired peripheral vasodilation, as evidenced by macro- and micro-vascular endothelial dysfunction (Joras and Poredos, 2008; Coutinho et al., 2011a; Grenon et al., 2014; Heinen et al., 2015), and increased arterial stiffness and pressure wave reflections in patients with PAD (Brewer et al., 2007; Amoh-Tonto et al., 2009; Coutinho et al., 2011b; Beckmann et al., 2015). This flow limitation results in inadequate O_2_ delivery to the mitochondria of contracting skeletal muscle, limiting oxidative phosphorylation (Brass and Hiatt, 2000; Pipinos et al., 2000, 2003).

### Skeletal muscle abnormalities

Although reduced blood flow is a significant contributor to clinical limb manifestations as indicated by the association of low ABI with leg pain symptoms (Wang et al., 2005), leg blood flow and ABI appear to be no more than weakly associated with functional/exercise capacity (Pernow and Zetterquist, 1968; Szuba et al., 2006; McDermott et al., 2013; Nardi Gomes et al., 2015). Indeed, revascularization of occluded blood vessels does not fully restore the muscle functional limitations in PAD patients (Regensteiner et al., 1993a; Gardner and Killewich, 2001; West et al., 2012), while exercise treatment improves functional capacity with negligible (Larsen and Lassen, 1966; Sorlie and Myhre, 1978) to modest increases in leg blood flow (Hiatt et al., 1990). These findings indicate that additional factors besides hemodynamic limitations contribute to the pathophysiology of the functional impairment in PAD.

The mismatch between oxygen and substrate demand and delivery to active skeletal muscles during exercise via a blood flow reduction (exercise-induced ischemia) and the associated prolonged hyperemic effect in the post-exercise resting period (reperfusion) trigger a cascade of pathophysiological responses (Hiatt et al., 2015). During ischemia and reperfusion, there is a rise in cytosolic Ca^2+^ with subsequent mitochondrial Ca^2+^ overload owing to mitochondria's role as an intracellular Ca^2+^ buffer, and there is a concurrent burst of reactive oxygen species (ROS) generation (Murphy and Steenbergen, 2008). These events can trigger mitochondrial permeability transition pore opening, which can limit adenosine triphosphate (ATP) production and activate apoptotic signaling, ultimately leading to cell death (Murphy and Steenbergen, 2008). Indeed, there is evidence of elevated ROS production and associated oxidative damage in skeletal muscle of PAD patients, which may be exacerbated by a compromised antioxidant defense system (Bhat et al., 1999; Pipinos et al., 2006, 2008). Most notably, previous studies in PAD patients demonstrated increased mitochondrial DNA injury (Bhat et al., 1999) as well as elevated protein oxidation and lipid peroxidation (Pipinos et al., 2006). Mitochondrial DNA damage has been detected in both limbs of patients with unilateral PAD, suggesting a potential systemic effect (Bhat et al., 1999). Inflammation and oxidant stress are likely implicated in subsequent skeletal muscle structural and metabolic abnormalities associated with PAD, including mitochondrial dysfunction, muscle fiber degeneration, muscle fibrosis, and muscle apoptosis and atrophy (Regensteiner et al., 1993b; Brass and Hiatt, 2000; Mitchell et al., 2007; Pipinos et al., 2007; Koutakis et al., 2015). In PAD patients with intermittent claudication, 3.8% of gastrocnemius cells were determined as apoptotic vs. 1.5% in age-matched controls, and caspase-3 activity (a key component of apoptosis which is activated by the mitochondrion) was double that in patients without PAD (Mitchell et al., 2007), suggesting that mitochondrial stress is linked to programmed cell death in skeletal muscle of PAD patients. The complex pathophysiology of exertional limb manifestations has been reviewed in detail by others (Pipinos et al., 2007, 2008; Hiatt et al., 2015).

## The role of the mitochondrion in PAD

**Figure 1:**
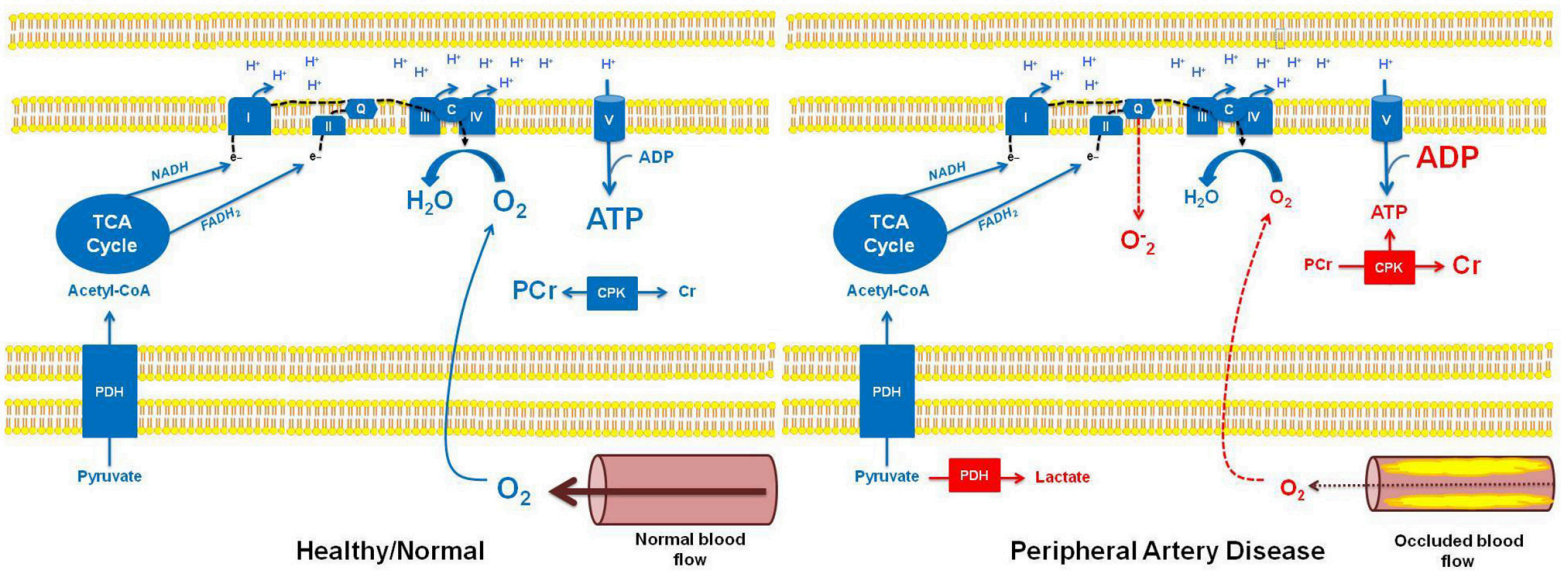
**Schematic overview of mitochondrial bioenergetics in healthy individuals with normal muscle blood flow, and in patients with peripheral artery disease (PAD) and occluded muscle blood flow**. In healthy individuals with normal muscle blood flow, pyruvate can undergo oxidation through pyruvate dehydrogenase (PDH), subsequently participating in the TCA cycle (as acetyl-CoA). Sufficient cellular O_2_ availability provides a terminal electron acceptor for the electron transport chain (ETC), ultimately allowing electron transfer and the generation of the electro-chemical potential needed for oxidative phosphorylation (ATP production). Further, electron flow to complex IV (IV) of the ETC prevents stagnation of electrons (e^−^) in the chain, thereby preventing superoxide (O^−^_2_) formation. In this setting, the creatine phosphokinase (CPK) reaction remains at a basal state of flux, where approximately two-thirds of the creatine (Cr) pool is stored as phosphocreatine (PCr). In contrast, reduced muscle blood flow in patients with PAD results in hypoxia and subsequent alterations in muscle bioenergetics. Specifically, in hypoxic tissue, pyruvate is unable to undergo an oxidative fate within the mitochondrion, instead being metabolized by lactate dehydrogenase in the cell cytosol, forming lactate. Similarly, reduced O_2_ availability in the mitochondria limits electron transfer and respiration resulting in electron accumulation in the ETC, which may lead to O^−^_2_ production at complex I and complex III, and subsequent oxidative stress. Importantly, reduced O_2_ availability and the subsequent impairment in oxidative phosphorylation in the muscle of patients with PAD will result in a reduction in ATP levels and a concomitant increase in ADP levels. This change in the cellular ATP to ADP ratio will drive the CPK reaction to breakdown PCr in order to buffer cellular ATP. This phenotype is most pronounced in muscle of patients with PAD when ATP turnover rates are higher, i.e., during muscle contraction associated with physical activity/exercise, and can lead to the localized muscle cramping and pain (claudication) experienced by individuals with PAD.

Mitochondria are the microscopic cellular combustion engines that utilize glucose and fat to provide our cells with ATP. Given its role in locomotion, skeletal muscle of the lower extremities is abundant with mitochondria. Mitochondrial oxidation of fuel substrates (glucose, fatty acids) converts to acetyl-CoA. The subsequent condensation of acetyl-CoA with oxaloacetate within the mitochondrial matrix forms citrate, initiating the tricarboxylic acid cycle (TCA). Generation of TCA cycle intermediates results in the reduction of electron carriers (NADH and FADH_2_), which in turn shuttle electrons to the electron transport chain of the inner mitochondrial membrane. Electron transfer results in the translocation of protons across the inner mitochondrial membrane, resulting in electro-chemical potential. This membrane potential is used by ATP synthase to phosphorylate adenosine diphosphate (ADP; i.e., oxidative phosphorylation; Mitchell, 1961). Critical to the process of oxidative phosphorylation is the requirement for a terminal electron acceptor, a role fulfilled in mitochondria by molecular O_2_. Thus, a patent arterial circulation is critical in providing cells and in particular their mitochondria with O_2_ to support mitochondrial respiration and thus oxidative phosphorylation (Figure 1).

As described above, the pathophysiology of the limb manifestations in lower extremity PAD is complex, and extends beyond the atherosclerotic occlusive disease and inadequate O_2_ delivery to the mitochondria of the working muscles. Since more than 90% of O_2_ is consumed within mitochondria (Rolfe and Brown, 1997), deficits in mitochondrial respiratory capacity and/or function may also contribute to limb manifestations in PAD patients. Early studies suggest incomplete oxidation of fuel substrates in PAD skeletal muscle biopsies as indicated by accumulation of metabolic intermediates (Brass and Hiatt, 2000; Pipinos et al., 2007; Hiatt et al., 2015). PAD gastrocnemius muscle displayed accumulation of acylcarnitines, which is suggestive of incomplete acyl-CoAs oxidation. Additionally, short-chain acylcarnitine accumulation was associated with impaired peak exercise performance (Hiatt et al., 1992). Muscle lactate concentrations have also been reported to be elevated in PAD patients, reflecting anaerobic glucose oxidation (Hiatt et al., 1992). While incomplete oxidation of substrates might be reflective of reduced mitochondrial oxidative capacity, it may also reflect adaptations in muscle that are independent of mitochondrial function, namely reduced blood flow.

## Assessment methods for skeletal muscle mitochondrial respiratory capacity and function

**Figure 2:**
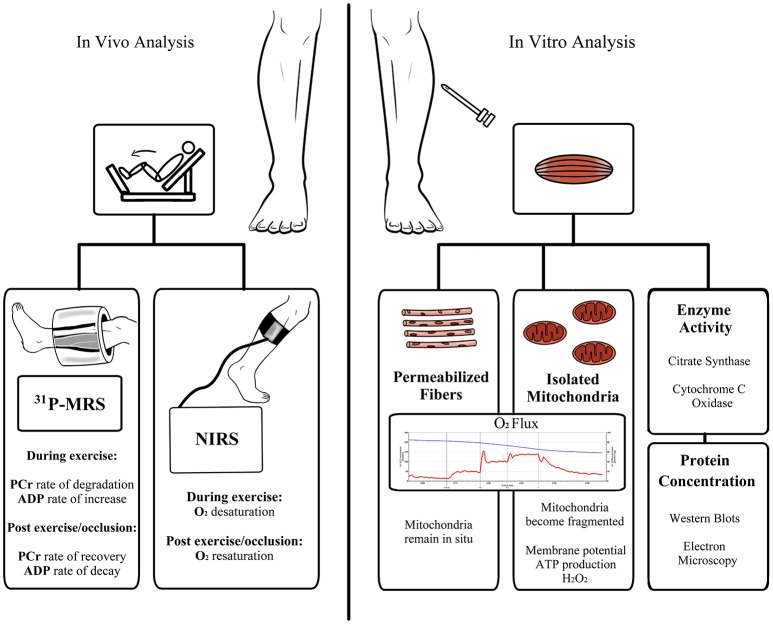
**Schematic overview of available methods for the assessment of skeletal muscle mitochondrial respiratory capacity and function**. Abbreviations: ADP, adenosine diphosphate; ATP, adenosine triphosphate; H_2_O_2_, hydrogen peroxide; NIRS, near-infrared spectroscopy; PCr, phosphocreatine; ^31^PMRS, ^31^Phosphorus magnetic resonance spectroscopy.

The numerous analytical techniques and read-outs of mitochondrial function can often lead to confusion as to the role of the mitochondrion in a given pathology. Thus, careful consideration in selecting appropriate analytical endpoints and interpretation of the data that these techniques provide is important if robust conclusions are to be made. Available techniques for measuring mitochondrial function (Figure 2) can be broadly categorized into: (i) *in vivo* measures of tissue oxidative capacity, (ii) *in vitro* determination of mitochondrial respiration in tissue samples or isolated mitochondria preparations, assaying ATP production rates, membrane potential, or ROS production in isolated organelles, or the quantification of mitochondrial protein abundance/enzyme activity. The pros and cons of these approaches are discussed in brief below.

Mitochondrial oxidative capacity can be assessed *in vivo* by ^31^Phosphorus Magnetic Resonance Spectroscopy (^31^PMRS; Hoult et al., 1974; Ackerman et al., 1980), a non-invasive method for the determination of relative concentrations of high-energy phosphate metabolites, principally phosphocreatine (PCr), and their kinetic changes during exercise (or ischemia) and their subsequent recovery rate constants. At the onset of intense exercise, PCr supplies ADP with its high-energy phosphate to form ATP, thereby buffering intracellular ATP levels, resulting in PCr degradation; the mean rate of PCr degradation during exercise provides an estimate of the failure of oxidative (plus glycolytic) ATP synthesis to meet ATP demand/turnover (Kemp et al., 1995b, 2001), with the initial rate of the PCr degradation being a measure of ATP turnover rate. During recovery following exercise, PCr and ADP return to baseline concentrations; the initial rate of PCr re-synthesis post-exercise serves as an estimate of the end-exercise rate of oxidative ATP synthesis (Blei et al., 1993).

Tissue oximetry by near-infrared spectroscopy (NIRS) offers a non-invasive approach for the assessment of the kinetics of (muscle) tissue O_2_ saturation and hemoglobin/deoxyhemoglobin levels (Jobsis, 1977). Analogous to PCr kinetics determined by ^31^PMRS, the rate of O_2_ desaturation in muscle during exercise reflects the rate of failure of O_2_ delivery to meet tissue O_2_ demand, and the post-exercise/occlusion recovery of O_2_ saturation rate correlates well with PCr recovery rate and provides an index of muscle respiratory capacity (Kemp et al., 2001; Nagasawa et al., 2003; Ryan et al., 2013).

Determination of muscle oxidative capacity *in vivo* offers several advantages. Firstly, oxidative capacity is determined under physiologic conditions. Further, measurements can be made non-invasively. While determination of PCr recovery can be costly, NIRS offers a more affordable alternative to highly costly MRI scanners, which correlates well with MRI-based approaches (Ryan et al., 2013) and muscle biopsy assessment of muscle respiratory capacity (Ryan et al., 2014). However, a caveat of these approaches is that while the capacity for oxidative phosphorylation can be quantified *in vivo*, it is difficult to discern whether a deficit in oxidative capacity results from impaired blood flow, reduced mitochondrial volume density, altered mitochondria quality, or a combination of these factors.

Biochemical analysis of muscle biopsy samples allows mitochondrial function to be assayed in tightly controlled systems. Typically, mitochondria can be isolated from muscle tissue by homogenization and centrifugation, or muscle fiber bundles can be chemically permeabilized and mitochondria studied *in situ*. For several decades, studying respiration or ATP production in isolated organelles was the gold-standard approach. However, mechanical isolation of mitochondria from muscle tissue liberates only ~50% of the total mitochondrial pool (Rasmussen et al., 2003). Further, more recent evidence has highlighted that the reticular structure and network formed by mitochondria *in situ* is significantly disrupted by isolation from skeletal muscle (Picard et al., 2011a,b). Importantly, this may lead to erroneous data concerning mitochondrial functionality (Picard et al., 2010). This may also explain the contradictory findings between mitochondrial respiratory capacity in isolated mitochondria and in muscle fiber bundles from PAD patients discussed in the following section. The development of high-resolution respirometry methodologies where mitochondrial respiration can be determined in permeabilized fiber preparations (Saks et al., 1998) now allows mitochondrial function to be determined when organelles remain in their normal architectural environment. In addition, the entire mitochondrial pool can be studied in ~5 mg (wet weight) of tissue, whereas much greater tissue volumes are typically required to isolate a viable pool of mitochondria. Biochemical approaches, such as high-resolution respirometry, allow for the determination of mitochondrial respiratory capacity and function in skeletal muscle samples (Kuznetsov et al., 2008; Gnaiger, 2009). Further, the function of specific complexes of the electron transport system can be determined. Collectively, such biochemical approaches allow detailed information to be generated on mitochondrial function. However, it should be noted that *in vitro* approaches typically employ supra-physiological O_2_ tensions and substrate concentrations. Clearly, these caveats need to be considered when interpreting data.

Finally, since assaying mitochondrial function in biopsy samples requires the use of specialized equipment and needs to be performed on fresh tissue, investigators often assay the protein levels and/or activity of mitochondrial enzymes spectrophotometrically as indicators of muscle oxidative function/capacity. These measurements can serve as valid surrogate markers of mitochondrial volume density and thus oxidative capacity. In particular, cardiolipin content and citrate synthase activity correlate well with electron microscopic determination of mitochondrial volume density in healthy adults (Larsen et al., 2012). Moreover, cytochrome C oxidase activity correlates with mitochondrial respiratory capacity (Larsen et al., 2012), at least in young healthy adults.

The various approaches described above allow different parameters of muscle mitochondrial performance to be determined, each having its own strengths and weaknesses. Due to the complementary nature of *in vivo* and *in vitro* measurements, a combination of approaches within a single study will likely offer the most comprehensive evaluation of skeletal muscle mitochondrial function.

## Skeletal muscle mitochondrial function in PAD

### Evidence of altered muscle oxidative capacity *in vivo*

A number of descriptive studies in small cohorts of PAD patients, primarily claudicants, have determined skeletal muscle oxidative capacity in PAD by means of ^31^PMRS or NIRS. The majority of these studies demonstrate increased PCr hydrolysis during exercise, and slower PCr recovery post-exercise in patients with PAD (Keller et al., 1985; Hands et al., 1986; Zatina et al., 1986; Wahl et al., 1994; Kemp et al., 1995a, 2001; Di Marzo et al., 1999; Pipinos et al., 2000; Greiner et al., 2006; Anderson et al., 2009). Greater PCr degradation during submaximal exercise reflects a greater mismatch between oxidative phosphorylation and ATP turnover. Slower PCr recovery post-exercise indicates reduced capacity for mitochondrial ATP production in PAD. To account for a confounding effect of exercise-induced ischemia and pH change (Kemp, 2004; Pipinos et al., 2007), PCr recovery was evaluated following mild isometric exercise by Pipinos et al. (2000) in order to minimize pH changes, which showed a similar slower PCr recovery in PAD patients compared to control, again suggesting reduced skeletal muscle oxidative capacity in patients with PAD. Collectively, these studies demonstrate that skeletal muscle oxidative capacity is diminished in PAD, where there is a greater reliance in substrate level phosphorylation to support cellular ATP demand. Additionally, prolonged PCr recovery was associated with poor treadmill performance but not with calf muscle perfusion in a cross-sectional study of 85 patients with mild to moderate PAD (mean ABI, 0.69), suggesting a role for reduced muscle oxidative capacity in the functional impairment in PAD, possibly independent of reduced blood flow (Anderson et al., 2009).

In support of PCr kinetic data, measurement of O_2_ kinetics by NIRS suggest increased muscle deoxygenation during exercise and slower reoxygenation post-exercise in PAD (Kooijman et al., 1997; Kemp et al., 2001; Egun et al., 2002; Comerota et al., 2003), where O_2_ recovery rate correlate well with PCr recovery rate in PAD patients (Kemp et al., 2001). Collectively, both MRS and NIRS provide robust evidence *in vivo* of impaired oxidative capacity in skeletal muscle of PAD patients. However, given the nature of these measurements, it is not possible to discern the underlying cause of reduced skeletal muscle oxidative capacity in PAD. For example, whether reduced oxidative capacity is the result of reduced mitochondrial volume density and/or diminished mitochondrial quality cannot be concluded from these *in vivo* measurements. Determination of mitochondrial function and protein abundance in biopsy samples is required to delineate potential deficits in mitochondrial density and/or quality in the metabolic myopathy that accompanies PAD.

### Evidence of altered mitochondrial function in skeletal muscle of PAD patients

A number of studies have attempted to determine skeletal muscle mitochondrial function in PAD patients (Jansson et al., 1988; Pipinos et al., 2003, 2006; Koutakis et al., 2015; van Schaardenburgh et al., 2016). In a series of studies, Pipinos and colleagues (Pipinos et al., 2003, 2006; Koutakis et al., 2015) evaluated mitochondrial respiratory capacity in saponin-permeabilized myofiber bundles from the gastrocnemius muscle of PAD patients using Clark-type oxygen electrodes. In the first of these studies, mitochondrial respiration was determined in skeletal muscle from 9 patients with advanced PAD (mean ABI, 0.4) and 9 PAD-free individuals (Pipinos et al., 2003). These authors found that ADP stimulated respiration supported by complex I was lower in PAD patients compared to controls, suggesting a quantitative deficit in mitochondrial respiratory capacity in PAD. Further, mitochondrial coupling control determined after the inhibition of adenine nucleotide translocase (via atractyloside titration) was lower in PAD vs. PAD-free individuals (Pipinos et al., 2003), suggesting altered mitochondrial quality (i.e., coupling control) in skeletal muscle of PAD patients.

In a consecutive study, Pipinos et al. (2006) combined respirometric and spectrophotometric measurements in gastrocnemius muscle biopsies of 25 advanced PAD (mean ABI, 0.34) and 16 PAD-free individuals to assess several parameters of muscle oxidative capacity. Mitochondrial respiration supported by electron transfer from complex I and complex II of the electron transport chain was assayed. Further, titration of mitochondrial inhibitors and specific electron donors was used to assay the respiratory capacity of complex III and complex IV of the electron transport chain. When normalized to citrate synthase activity, the investigators reported lower ADP-stimulated respiration supported by complex I in PAD patients, but no differences between groups in respiration supported by complex II, suggesting substrate specific deficits in respiratory capacity of muscle mitochondria of PAD patients. Moreover, these authors reported lower respiratory capacity at the level of complex III and complex IV, which likely reflect an overall reduction in mitochondrial respiratory capacity in muscle of PAD patients. In support of these respirometric data, spectrophotometric assays of mitochondrial enzyme activities normalized to citrate synthase activity demonstrated lower NADH dehydrogenase (complex I), ubiquinol cytochrome c oxidoreductase (complex III) and cytochrome c oxidoreductase (complex IV) activities in muscle of PAD patients, where complex I and III enzyme activities correlated with complex-I-supported respiration and complex-III-supported respiration, respectively (Pipinos et al., 2006). In agreement with the above findings, Brass et al. (2001) demonstrated lower NADH dehydrogenase activity when normalized to citrate synthase activity in gastrocnemius muscle biopsies from 17 PAD (mean ABI, 0.64) patients compared with 9 controls. However, these authors found no significant differences in mitochondrial enzyme activities of other electron transport chain complexes. These contrasting results may relate to disparate control groups and/or severity of PAD between studies (advanced vs. moderate ABI values).

More recently, the group led by Pipinos (Koutakis et al., 2015) provided further evidence of skeletal muscle mitochondrial dysfunction in PAD. Studying 30 PAD (mean ABI, 0.55) and 30 PAD-free patients, the authors reported significantly lower ADP-stimulated respiration supported by complex I and complex IV-dependent respiration normalized to citrate synthase activity in PAD patients vs. controls (Koutakis et al., 2015). In contrast to their previous report (Pipinos et al., 2006), PAD patients did not have diminished complex III-dependent respiration. Once again, this possibly pertains to differences in the characteristics of the control groups between studies, with the control group being closely matched to the PAD group in the latest study (Koutakis et al., 2015) compared with a relatively healthier control group in the prior study (Pipinos et al., 2006). Furthermore, fluorescent microscopy data revealed an irregular, uneven and patchy distribution of mitochondria in PAD gastrocnemius myofibers, with absence of mitochondria in desmin-dense myofiber areas that correlated with decreased complex I- and IV-dependent respiration. In addition, this group has reported increased protein carbonyl and 4-hydroxy-2-nonenal (4-HNE) contents in gastrocnemius myofibers in PAD patients compared to PAD-free individuals, across all myofiber types (Pipinos et al., 2006; Weiss et al., 2013; Koutakis et al., 2014), indicating elevated oxidative stress in muscle of patients with PAD. Increased oxidative damage in muscle samples from these PAD patients was associated with reduced myofiber size, and clinical disease progression (Weiss et al., 2013; Koutakis et al., 2014). Type II (fast-twitch) and I/II fibers (mixed) myofibers had higher carbonyl content (i.e., greater oxidative damage) and displayed a greater reduction in size compared to Type I fibers (slow-twitch) in PAD vs. PAD-free gastrocnemius muscle. The oxidative damage and reduced fiber size is also coupled to a shift from Type II myofibers to Type I and Type I/II fibers (Koutakis et al., 2014). These findings suggest that oxidative stress and changes in the mitochondrial structure and architecture may contribute to the lower respiratory capacity of skeletal muscle from PAD patients. Furthermore, data from a recent study by White et al. (2016) is suggestive of impaired clearance of damaged mitochondria despite greater activation of mitophagy in gastrocnemius muscle biopsies from PAD patients, which may influence mitochondrial turnover rates.

Besides the observations in patients, evidence from studies using a mouse model of hindlimb ischemia indicates that disruption of mitochondrial detoxifying/antioxidant systems may partially account for compromised mitochondrial respiration and skeletal muscle abnormalities in PAD patients. In these studies, mitigation of mitochondrial oxidative stress by mitochondrial-targeted therapy improved mitochondrial function (Ryan et al., 2016a,b). In addition, mice deficient in mitochondrial aldehyde dehydrogenase 2, an enzyme responsible for toxic aldehyde clearance (such as 4-HNE), exhibited greater gastrocnemius muscle atrophy in response to chronic hindlimb ischemia compared to wild-type mice (Liu et al., 2015), further supporting a role for oxidative stress in the mitochondrial myopathy accompanying PAD.

It should be noted that while measurement of citrate synthase activity correlates well with mitochondrial volume density in healthy adults (Larsen et al., 2012), whether this is true in PAD patients has not been confirmed. Several studies suggest no significant differences in mitochondrial volume density as indicated by citrate synthase activity in the gastrocnemius muscle between PAD and PAD-free patients (Bhat et al., 1999; Wang et al., 1999; Brass et al., 2001; Hou et al., 2002; Pipinos et al., 2006). However, other studies report higher citrate synthase activity in gastrocnemius muscle of the claudicant leg than the asymptomatic leg in patients with unilateral PAD or between legs of different PAD severity (Jansson et al., 1988; Hiatt et al., 1996), in line with mitochondrial volume density data determined by electron microscopy (EM) in anterior tibial muscle of unilateral PAD patients (Angquist and Sjöström, 1980). Yet, a more recent study has demonstrated reduced mitochondrial volume density by EM in the vastus lateralis of 14 claudicants (mean ABI, 0.73; Baum et al., 2016). Differences in muscle sampling sites, severity of PAD, or methodologies used may account for the disparate findings between studies. Non etheless, muscle citrate synthase activity was recently identified as a predictor of mortality rate in PAD patients, with mid-range values being associated with greater survival (Thompson et al., 2015).

While the current literature is somewhat conflicting, the above data suggest reduced muscle mitochondrial respiratory capacity in PAD, which is likely accompanied by a change in the mitochondrial volume density, at least in the more severe manifestations of the disease. However, there is a paucity of data on skeletal muscle mitochondrial coupling and flux control in patients with PAD. Early work by Elander et al. (1985) studied both mitochondrial respiratory capacity and coupling control in isolated mitochondrial sub-populations from the gastrocnemius muscle of PAD patients (mean ABI, 0.58) and PAD-free controls. Contrary to the more recent evidence suggesting impaired mitochondrial respiration, these authors reported higher respiration supported by complex I and respiratory control ratio for complex I and II in specific isolated mitochondrial sub-populations of PAD vs. PAD-free patients. The discrepant findings may reflect certain limitations with studying mitochondrial respiration in isolated mitochondria than in permeabilized muscle fibers, such as low yields and disruption of the mitochondrial network (Picard et al., 2010, 2011a,b). Indeed, van Schaardenburgh et al. (2016) recently studied both mitochondrial respiratory capacity and coupling control in permeabilized muscle fibers from the gastrocnemius muscle of 11 patients with PAD (mean ABI, 0.65) and 11 PAD-free healthy older adults. They found lower mitochondrial respiration supported by complex I but normal complex-II-supported respiration in PAD vs. PAD-free patients, in contrast to the previous findings of Elander et al. (1985). However, when normalized to citrate synthase activity, mitochondrial respiration ceased to differ between PAD and PAD-free patients, even though citrate synthase activity values *per se* did not differ between groups. Yet, the coupling control efficiency (similar to respiratory control ratio) for complex I was 2.5 times lower in PAD vs. healthy older adults, which may serve as an index of intrinsic mitochondrial dysfunction at complex I. Moreover, the coupling control factor (and index of respiratory control) for complex II was greater in PAD patients vs. controls, perhaps suggesting a compensatory response to impaired complex I function. Here, it should be noted that this study (van Schaardenburgh et al., 2016) was designed to examine the acute effects of exercise on mitochondrial respiration within groups separately, possibly explaining why the groups were not age-matched, and thus, any between group baseline comparisons should be interpreted with caution. Yet, these recent findings (van Schaardenburgh et al., 2016) provide valuable insight in mitochondrial quality and offer the basis for future studies to comprehensively characterize skeletal muscle mitochondrial function in PAD.

### Existing therapeutic approaches to restore mitochondrial function and skeletal muscle abnormalities in PAD

Exercise training, in particular supervised exercise therapy, is recommended for PAD (Fokkenrood et al., 2013; Rooke et al., 2013), for its beneficial effects on functional capacity (McDermott et al., 2009). A limited number of studies in patients with PAD have highlighted the potential of exercise therapy to improve skeletal muscle metabolism and mitochondrial function. In 10 PAD patients, improved exercise performance with supervised exercise training was associated with improved lipid oxidation (as indicated by altered carnitine metabolism; Hiatt et al., 1990, 1996). Supervised exercise training resulted in an increase in pyruvate- and L-malate-induced mitochondrial respiration of calf muscle from 8 PAD patients compared to 7 untrained patients and 11 healthy controls as demonstrated by *in vitro* approaches (Hou et al., 2002). Furthermore, a pilot study has suggested improved PCr recovery kinetics indicating enhanced oxidative capacity in PAD patients following supervised exercise training, but no comparisons were made to a control group (Brizendine et al., 2014). Pentoxifylline, an FDA approved vasoactive drug (phosphodiesterase inhibitor) for intermittent claudication, has been shown to improve oxidative capacity assessed by PCr recovery kinetics, which in turn is associated with improved functional capacity in patients with PAD (Pipinos et al., 2002).

A limited number of studies have also investigated the effects of revascularization procedures on mitochondrial function with inconsistent results between studies. Improved but not complete restoration of oxidative capacity determined by PCr recovery kinetics in lower extremity muscles has been demonstrated in PAD patients who underwent lower extremity revascularization procedures (Schunk et al., 1998; West et al., 2012). In a third study, treatment of PAD patients with lower limb PTA or bypass surgery failed to demonstrate significant improvement in oxidative metabolism determined by PCr recovery kinetics in calf muscle, albeit normalization of hemodynamic parameters (Zatina et al., 1986). Collectively, clear data on the role of surgical revascularization in improving skeletal muscle oxidative capacity in PAD patients is lacking. In contrast, the majority of data on exercise training suggests efficacy in terms of restoring muscle oxidative capacity in PAD patients. Thus, one may conclude that reduced muscle mitochondrial function in PAD may be influenced more directly by reduced muscle contraction, underscoring the importance of physical activity and exercise training strategies in the management of PAD. Since oxidative stress may be responsible for some of the deficits in muscle mitochondrial function observed in patients with PAD, therapies targeting mitochondrial antioxidant systems may hold therapeutic value. Indeed, targeted antioxidant therapy restores mitochondrial function in a rodent model of hindlimb ischemia (Ryan et al., 2016a,b). The therapeutic value of manipulating mitochondrial antioxidant systems as well as other mitochondrial quality control mechanisms, including mitochondrial dynamics (fusion-fission balance), and mitochondrial turnover (mitophagy) in the context of PAD has been comprehensively reviewed elsewhere (Ueta et al., 2017).

Although this review focuses on the role of mitochondria in the myopathy of lower extremities PAD, additional factors also contribute to the development of PAD. Frequently, these processes are the result of an orchestrated response to ischemia and involve numerous cell types. In the gastrocnemius muscle of PAD patients, vascular smooth muscle cells have been found to shift to a pro-fibrotic phenotype, expressing transforming growth factor-beta 1 (TGF-β1; a pro-fibrotic cytokine; Ha et al., 2016). Elevated TGF-β1 expression was associated with accumulation of fibroblasts and collagen deposition in the muscle biopsies from these PAD patients. As PAD progresses, collagen deposition expands from the perivascular area and infiltrates the gastrocnemius myofibers (Ha et al., 2016). Further, in the ischemic microenvironment, endothelial and skeletal muscle cells produce angiogenic factors, such as vascular endothelial growth factor (VEGF) and angiopoietin-1. *In vitro* data suggest that VEGF and angiopoietin-1 secreted by skeletal muscle and endothelial cells in response to ischemia may play a role in muscle remodeling (McClung et al., 2015). In addition, rodents with hindlimb ischemia show improved muscle regeneration when treated with angiogenic and myogenic growth factors (Borselli et al., 2010). Hence, targeting the multicellular skeletal muscle environment may be of therapeutic value in the management of the myopathy associated with PAD.

### Summary and future directions

Collectively, current evidence from *in vivo* and *in vitro* methodologies suggests reduced skeletal muscle oxidative capacity in PAD. Diminished skeletal muscle oxidative capacity appears to result from both impaired blood flow and altered mitochondrial respiratory capacity and quality. Although these findings are important, they are the product of a number of small and sometimes disparate exploratory studies, which do not allow for firm conclusions to be drawn regarding the role of mitochondrial function in PAD. The tight coupling between the vasculature, oxygen delivery, and muscle mitochondrial respiration constitutes a dynamic system of complex interactions that may be best explored concurrently with a combination of *in vivo* and *in vitro* methodologies. In particular, we suggest future studies to aim to: (i) be sufficiently-powered; (ii) use appropriate controls to draw comparisons to by matching PAD and control groups on potential confounders, such as age, gender, weight, smoking status, coronary artery disease, diabetes, dyslipidemia, and hypertension; (iii) stratify PAD based on severity/clinical manifestations (ie., asymptomatic, claudication, atypical, critical limb ischemia); (iv) integrate a combination of methodologies in order to assess oxidative capacity *in vivo*, determine intrinsic mitochondrial respiratory capacity, and coupling control ratios (mitochondrial quality), and muscle mitochondrial volume density in muscle biopsies of the same patients; (v) determine the relationships between mitochondrial function, clinical PAD parameters, and functional limitations which was not feasible in the majority of prior studies due to being relatively underpowered. Inclusion of patients with unilateral PAD and assessments on both limbs would also be highly informative. Moreover, analysis of mitochondrial function in biopsies collected from different regions of the affected muscle in the same patient will provide additional important information on the nature of mitochondrial dysfunction in muscle from PAD patients. Furthermore, data on the long-term effects of revascularization procedures on mitochondrial function is currently lacking. Finally, transcriptome-wide analysis of muscle from patients with PAD would be useful and may identify new therapeutic targets worthy of further investigations.

## Conclusion

The lack of a clear understanding of the role of mitochondrial dysfunction in PAD represents a significant roadblock in the development of novel strategies to restore muscle function, ameliorate limb symptoms, and improve functional capacity in PAD patients. Therefore, generation of new data concerning the role of bioenergetics in PAD may contribute to the development of novel therapies aimed at reducing morbidity in patients living with PAD. Considering the rising prevalence of PAD, the persistence of PAD-associated myopathy after revascularization, its functional and economic impact, and the limited therapeutic options that currently exist, further research in this field is warranted.

## Author contributions

Substantial contribution to the concept and interpretation of available evidence (All authors); Drafted the work (VR and CP). Revising it critically for important intellectual content (ON, GF, ZC, and BR). Final approval of the version to be published (All authors). Agreement to be accountable for all aspects of the work in ensuring that questions related to the accuracy or integrity of any part of the work are appropriately investigated and resolved (All authors).

## Funding

Seed funding for this project was supplied by the Department of Surgery, UTMB. This work was also supported by the following grants: R56 AG051267, P30 AG024832 and SHC-84090, and was conducted with the support of the Institute for Translational Sciences at UTMB, supported in part by a Clinical and Translational Science Award (UL1TR000071).

### Conflict of interest statement

The authors declare that the research was conducted in the absence of any commercial or financial relationships that could be construed as a potential conflict of interest.
